# Bibliometric analysis of Ewing sarcoma from 1993 to 2022

**DOI:** 10.1186/s12885-023-10723-7

**Published:** 2023-03-24

**Authors:** Guangtao Han, Ting Liu, Pengde Kang

**Affiliations:** Department of Orthopaedics Surgery, West China Hospital, Sichuan University, Chengdu, China

**Keywords:** Ewing sarcoma, Bibliometric analysis, CiteSpace, VOSviewer

## Abstract

**Background:**

Ewing sarcoma has attracted more attention in recent years but has yet to be bibliometrically analyzed. Hence, this study investigated the trend of Ewing sarcoma over the past 30 years with bibliometric analysis.

**Methods:**

Original publications related to Ewing sarcoma were obtained from the Science Citation Index Extension (SCI-E), Social Sciences Citation Index (SSCI), and Web of Science Core Collection (WoSCC) between 1993 and 2022. CiteSpace and VOSviewer were used to extract the countries/regions, institutions, authors, journals, references, and keywords involved in this topic to identify and analyze the research hotspots and trends in this field.

**Results:**

Over the past 30 years (especially in the past five years), the number of articles published on Ewing sarcoma continued to increase, and the most published country was the United States of America (USA). High-frequency keywords included "Ewing sarcoma", "tumor", "family", "bone", "chemotherapy", "expression", "primitive neuroectodermal tumor", "prognostic factors", "children", and "survival rate". According to the analysis of keyword saliency of Ewing sarcoma, we found that "chromosome translocation", "intergroup", "sarcoma", "genomic landscape", and "children oncology group" were emerging research hotspots. The timeline of the cluster map of co-cited literature indicated that the treatment of Ewing sarcoma emerged as a research hotspot.

**Conclusion:**

Researchers' understanding of Ewing sarcoma has improved dramatically over the past 30 years. At present, the research hotspots of Ewing sarcoma mainly focus on the aspects of "chromosome translocation", "intergroup", and "sarcoma". In addition, the timeline of the cluster map of co-cited literature indicated the emergence of the treatment of Ewing sarcoma as a research hotspot.

## Introduction

Ewing sarcoma ranks second among orthopedic malignant tumors, behind osteosarcoma [1]. It is common in adolescents, and the incidence rate accounts for 10 to 15% of orthopedic tumors [2]. The peak incidence occurs at the age of 15, and the incidence rate in males is higher than in females, with a ratio of 3:2 [3]. Although Ewing sarcoma accounts for only 1% of human malignant tumors, it is extremely aggressive and can rapidly metastasize to the lung and other tissues. The five-year overall survival rate of patients with both Ewing sarcoma and a localized disease is between 65 and 75% [4]. The common symptom of Ewing sarcoma is intermittent pain in children and adolescents, which may complicate and delay the diagnosis process and is often neglected. The survival rate of patients is approximately 70% after five years and decreases sharply to 30% after 10 years. Tumor metastasis at the time of diagnosis further reduces the survival rate after five years to only 25% [3]. The poor survival rate of Ewing sarcoma has since gained awareness.

Bibliometric analysis is a method of using mathematical and statistical techniques to analyze books, articles, and other literature for a general understanding of a new field. It is also a tool to explore the structure and trends of a topic through visualization and statistics for a quantitative assessment of the impact of research literature on selected research areas, countries/regions, research collaborations, journals, institutions, and authors in a given period [5, 6]. Compared with traditional systematic reviews and meta-analyses, bibliometric analysis can reveal key issues and developments in the field of interest more systematically and intuitively, thereby guiding future research [7]. CiteSpace is a visual analysis software that studies the structure, patterns, and distribution of research areas [8]. VOSviewer software is effectively used for knowledge domain mapping [9]. VOSviewer and CiteSpace can directly reflect the development of the research field by providing a large amount of data, including the productivity of authors and institutions, geographical distribution by region, and the results of collaborative relationships, and they are widely used in a variety of application areas [8, 9].

Bibliometrics and visualization methods have been used for osteosarcoma, osteoarthritis, osteonecrosis of the femoral head, and other orthopedic diseases. However, a review of Ewing sarcoma using bibliometrics and visualization methods has not been published to investigate the longitudinal and transverse characteristics of Ewing sarcoma, trends, and its multiple branches. Hence, this study explored the major contributors to the field over the past two decades and identified the hotspots and research trends in various aspects. The results of this study provided new insights on Ewing sarcoma to global research teams and orthopedic specialists.

## Materials and methods

### Data collection and search strategy

**Fig. 1:**
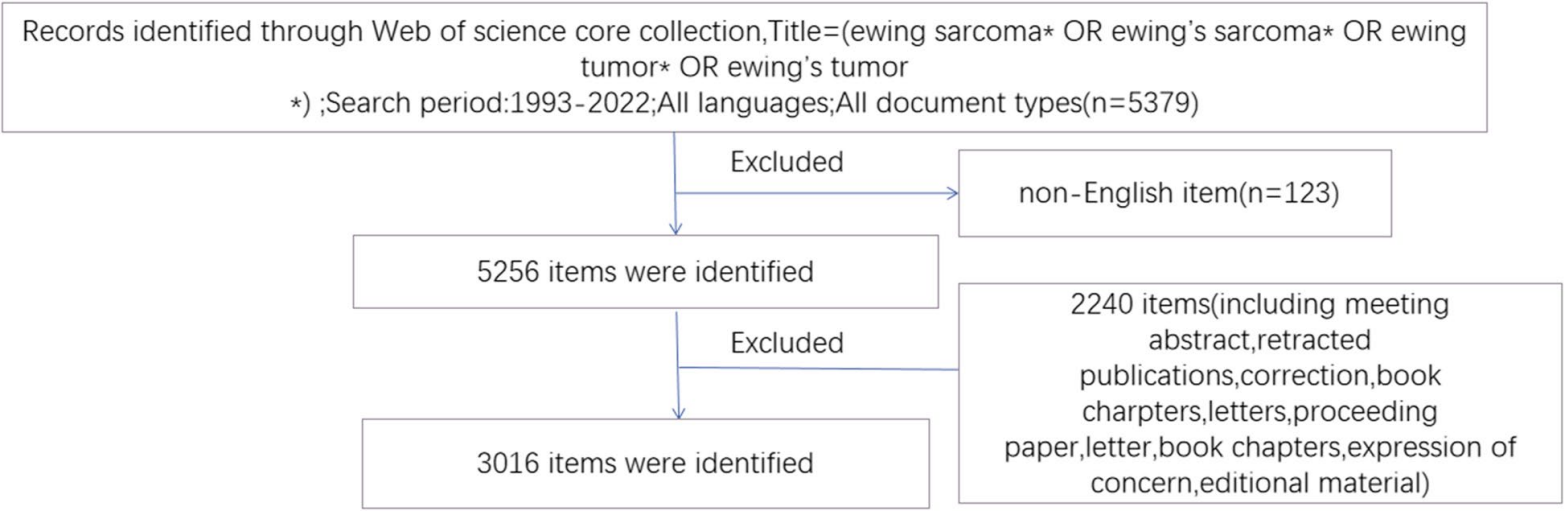
Article selection process

In this study, bibliometric analysis was performed using the Science Citation Index Extended Edition and the Social Sciences Citation Index of the WoSCC database. The search phrase was: Title = (Ewing sarcoma* OR Ewing’s sarcoma* OR Ewing tumor* OR Ewing’s tumor*). The language of the document was limited to English. Relevant publications between 1993 and 2022 were searched from the database, and only original articles and reviews were included in our analysis. Conference abstracts, editorial material, correspondence, and book chapters were excluded. The detailed flow chart of the article selection process in this study is displayed in Fig. 1. To avoid bias from database updates, all publication searches and file downloads were conducted on September 21, 2022. Two researchers in the study independently examined data collection and input. The differences in the results obtained by the two researchers were resolved through discussion or consultation with experts in the field to reach a consensus.

### Bibliometric analysis

Data was converted into text documents before uploading into the bibliometric analysis software. CiteSpace 6.1 R3, 64-bit (Drexel University, Philadelphia, PA, USA), VOSviewer 1.6.11(Leiden University, the Netherlands), and the Bibliometrics Online Analysis platform (http://bibliometric.com/) were used to locate the network characteristics of co-cited references, keywords, countries, institutions, authors, journals and "keyword burst" and visually present the results. The 2022 journal citations report was analyzed, including its impact factor category quartiles, and category ranking.

VOSviewer can be used to construct "scientific knowledge networks" that map the evolution of research fields and institutional collaborations and predict future research hotspots. In this work, VOSviewer was used to visually estimate word co-occurrence and construct density maps. The co-occurrence analysis function in VOSviewer can be used to classify keywords into different categories, which are represented by different colors. Cluster analysis of research hotspots can be visualized, and the keyword co-occurrence network can predict the growth trend.

A series of publication analyses were performed using CiteSpace to identify research hotspots for Ewing sarcoma. The analysis included publications, co-cited references, and relevant keywords. In the constructed network visualization, nodes reflect observed items, with larger nodes representing items that occur more frequently. In addition, CiteSpace was used to analyze centrality, a metric that defines the importance of network nodes, where more prominent nodes represent higher centrality. Centrality is used to measure the importance of a node's position in a network. The higher the centrality, the more connections in the network that pass through that node.

## Results

### Overview of publications

**Fig. 2:**
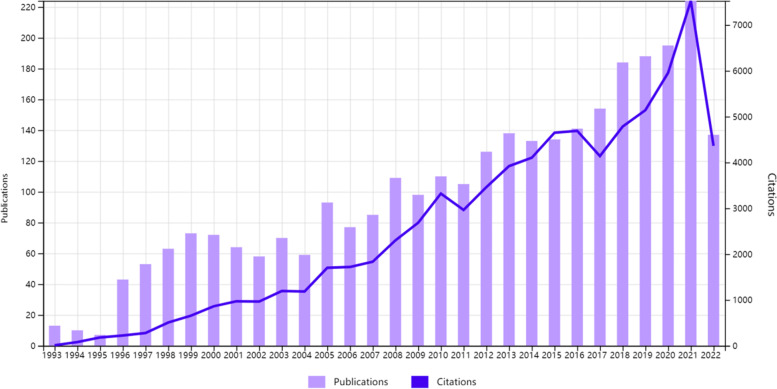
The number of publications on Ewing sarcoma research from 1993 to 2022

**Fig. 3:**
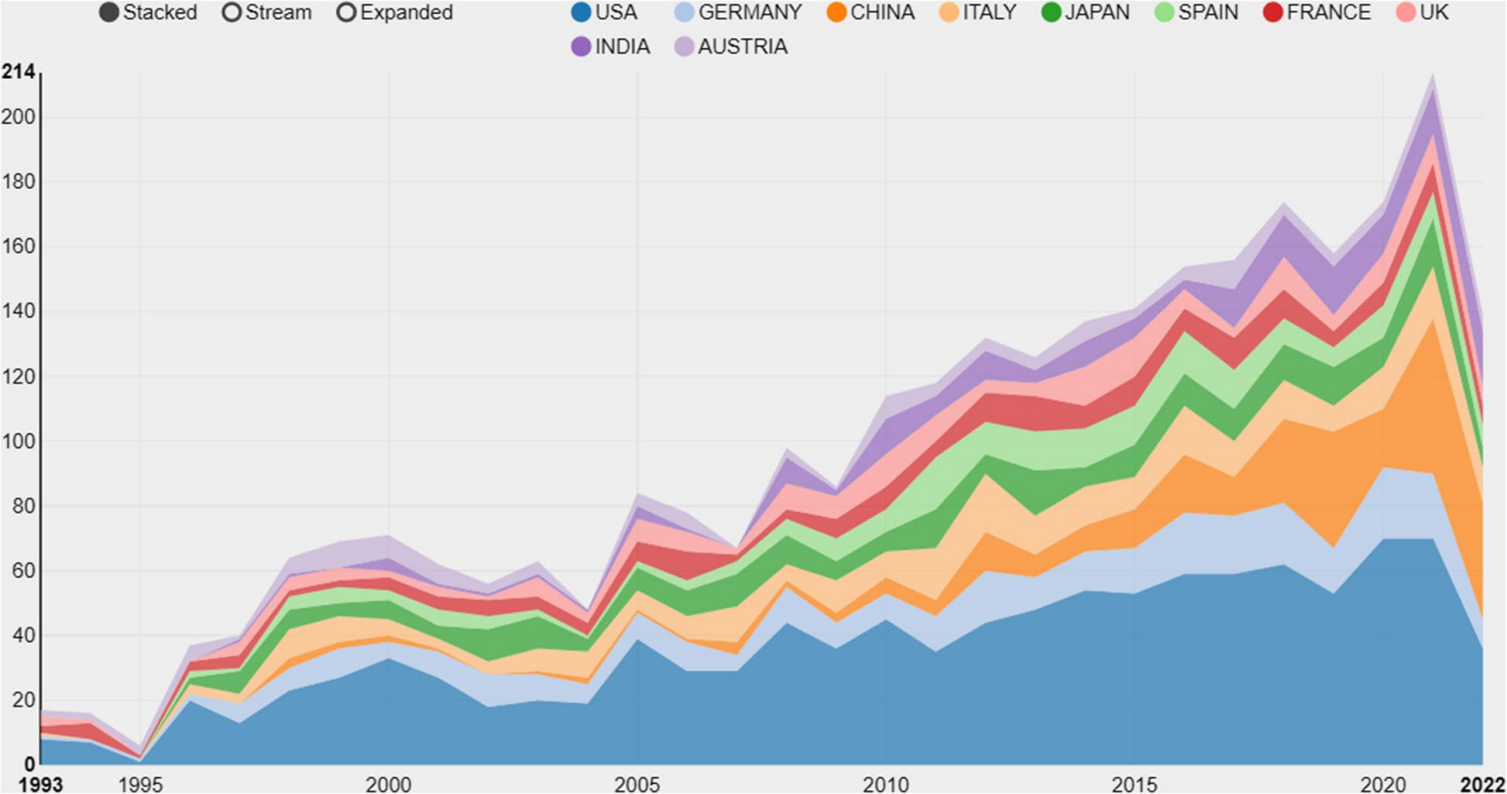
Top 10 countries/regions with Ewing sarcoma research from 1993 to 2022. Different colors denote different countries

From 1993 to 2022, a total of 3016 original articles on Ewing sarcoma were published. Over the past 30 years, there has been an overall increase in the number of studies related to Ewing sarcoma (Fig. 2). At the same time, the magazines publishing about Ewing sarcoma also demonstrated a growing trend, with the highest increment recorded between 2015 and 2021. Ewing sarcoma-related articles were published the most in 2021, accounting for about 7% of all original articles on Ewing sarcoma (Fig. 3).

### Distribution of countries/regions and institutions

**Fig. 4:**
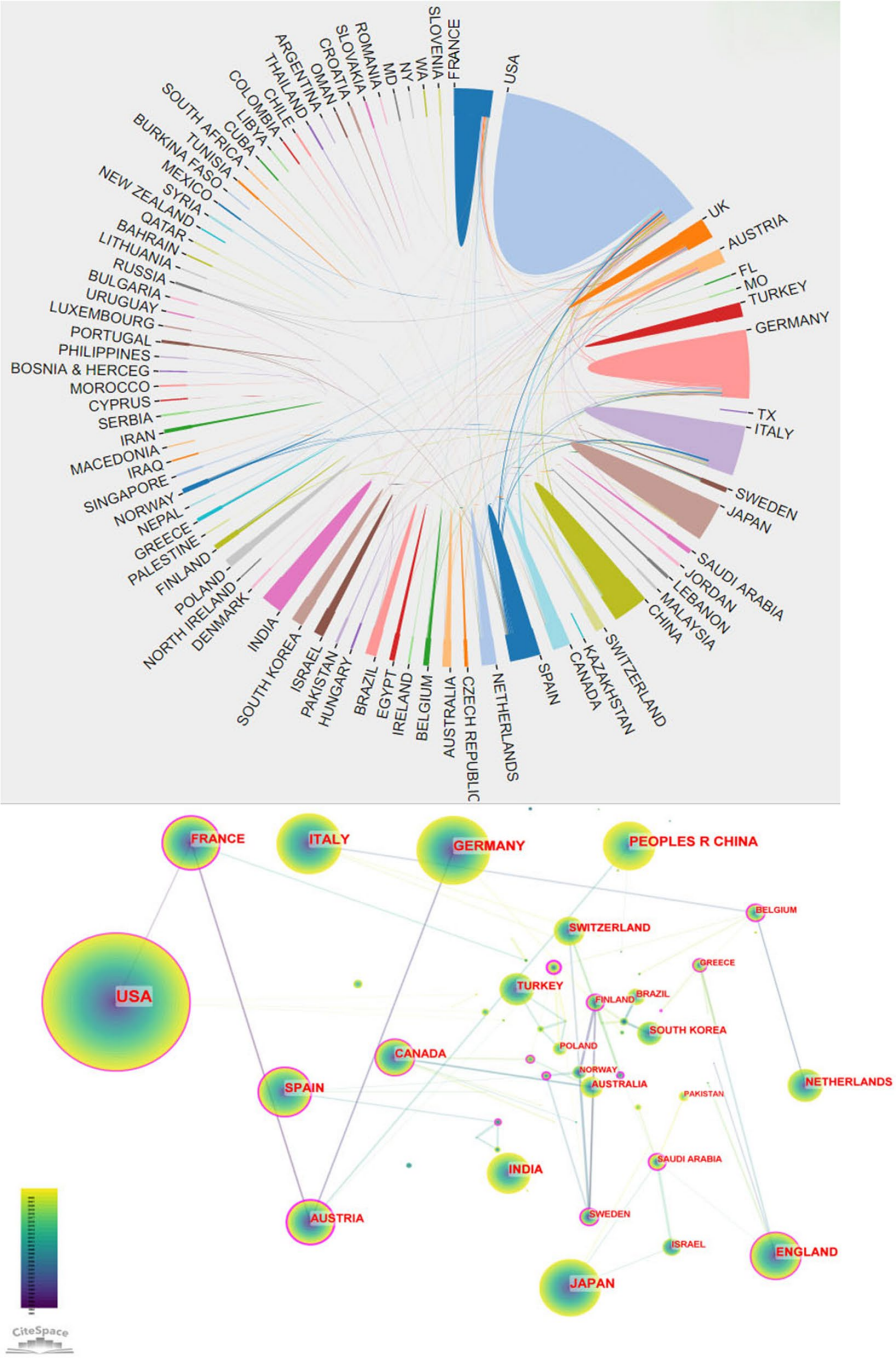
Cooperation of countries on Ewing sarcoma research publications from 1993 to 2022. Different colors denote different countries. The size of each node represents the frequency of node occurrence. High centrality nodes with purple rings indicate a turning point or pivot point in the domain. Centrality is used to evaluate the importance of a node in the network, where centrality corresponds with the number of links passing through the node

Figure 4 illustrates the national collaboration network for Ewing sarcoma research. All the data were analyzed in CiteSpace, and the results are displayed in Table 1. The USA had the largest number of Ewing sarcoma-related articles, with 1082 articles, followed by Germany with 298 articles, Italy with 252 articles, China with 249 articles, Japan with 220 articles, Spain with 181 articles, France with 167 articles, India with 155 articles, England with 150 articles, and Austria with 122 articles. The USA accounted for about 35.9% of articles (Table 1). Centrality is an index that defines the importance of network nodes, where more prominent nodes represent higher centrality. In the study of Ewing sarcoma, the USA had the highest centrality (0.39), followed by Germany (0.18) and England (0.17) (Table 1). In cooperative networks, higher centrality represents closer cooperation. The country-based research network map displayed a lower density, indicating the relative independence of the research teams and the need for further collaboration.

**Table 1:**
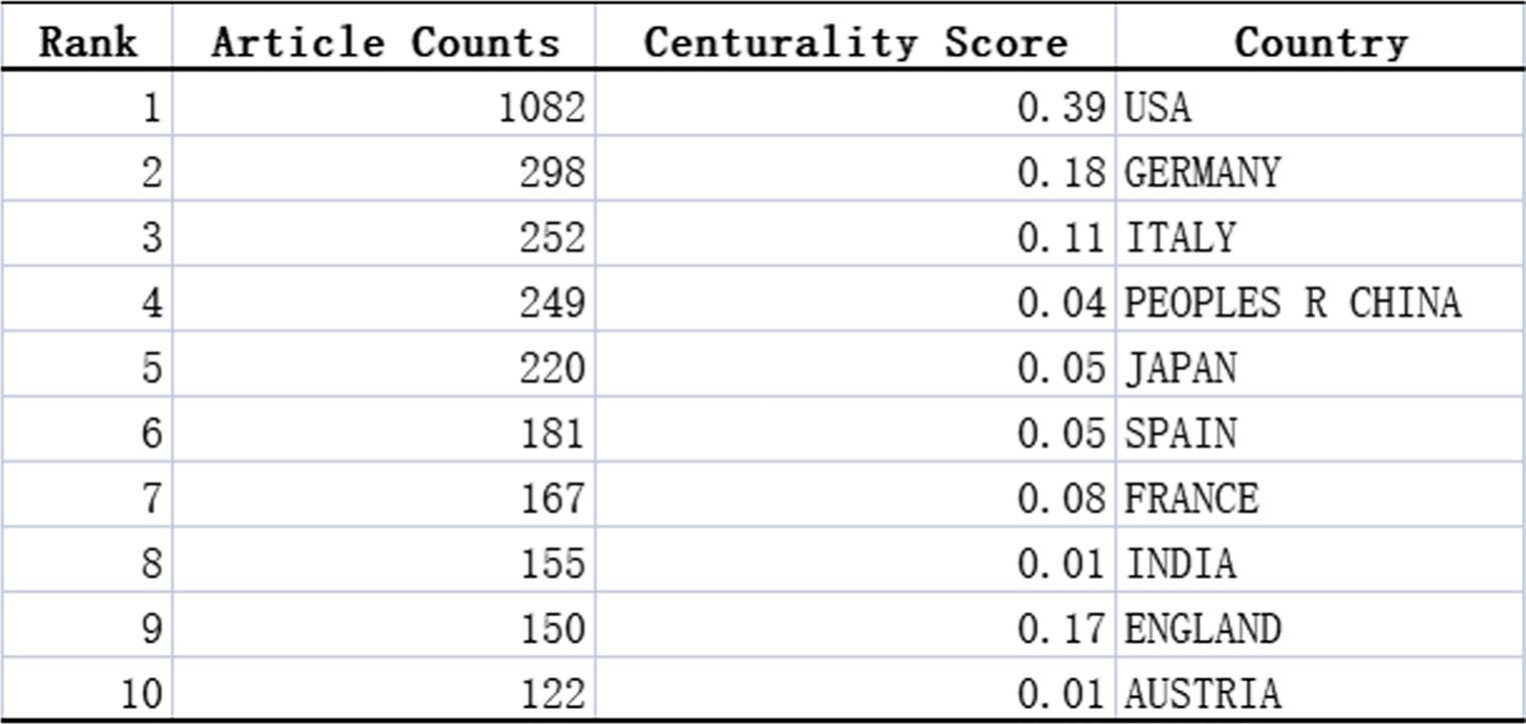
Top 10 countries with the most studies on Ewing sarcoma from 1993 to 2022

**Fig. 5:**
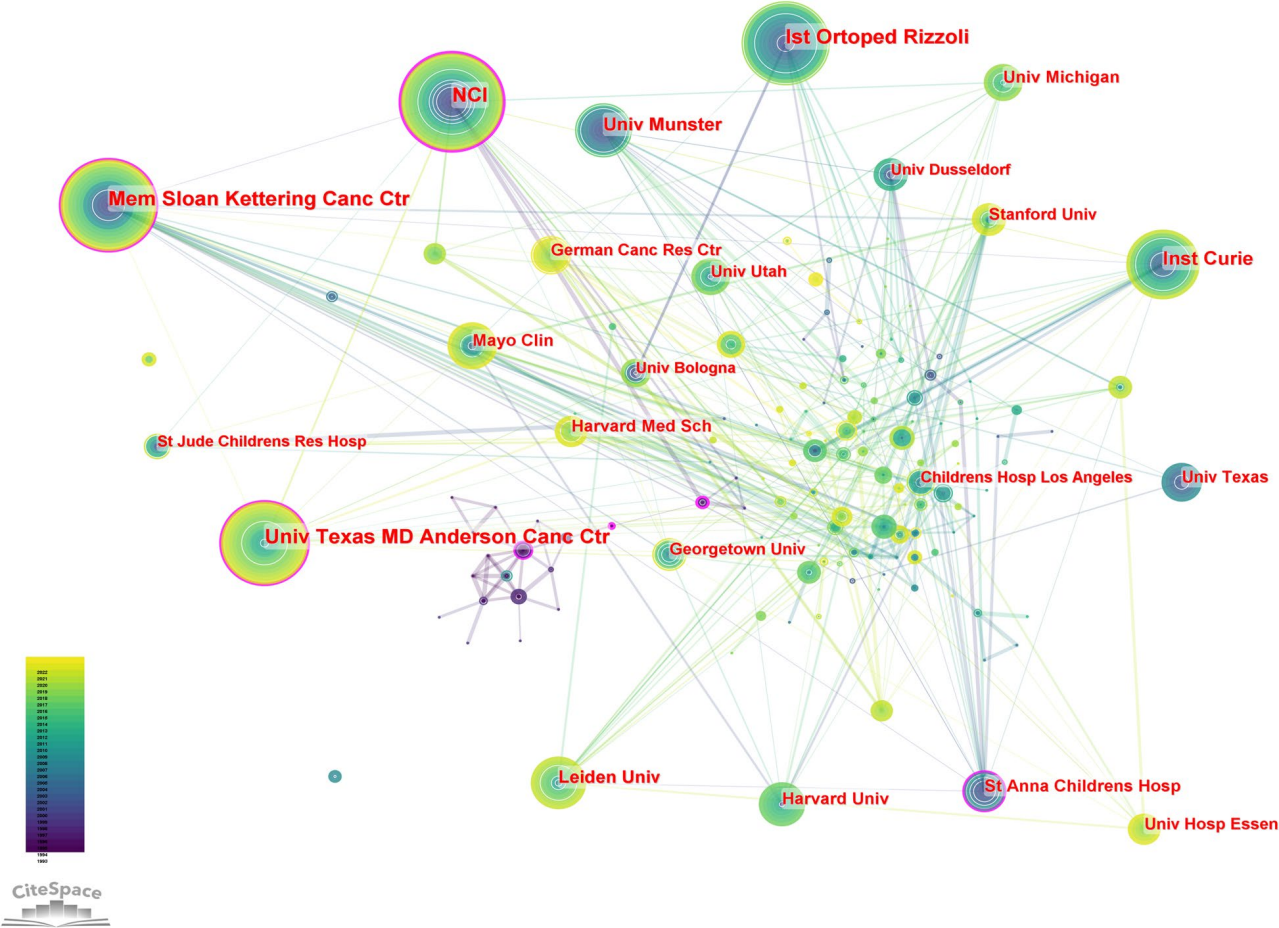
Cooperation of collaborations relating to Ewing sarcoma research publications from 1993 to 2022. The size of each node represents the frequency of node occurrence. High centrality nodes with purple rings indicate a turning point or pivot point in the domain. Centrality is used to evaluate the importance of a node in a network, where centrality corresponds with the number of links passing through the node

Similarly, the top five institutional cooperation included NCI (73), Mem Sloan Kettering Canc Ctr (70), Univ Texas MD Anderson Canc Ctr (70), Ist Ortoped Rizzoli (66), and Ist Curie (49) (Table 2). Among these, NCI centrality was the highest at 0.31, followed by Mem Sloan Kettering Canc Ctr (0.20) and Univ Texas MD Anderson Canc Ctr (0.11) (Fig. 5; Table 2). The low centrality of all institutions indicated low inter-agency cooperation.

**Table 2:**
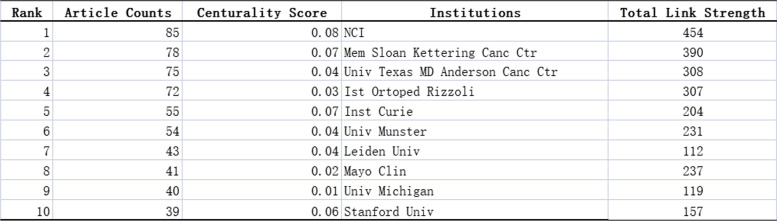
Top 10 collaborations with the most studies on Ewing sarcoma from 1993 to 2022.

### Author network analysis

**Fig. 6:**
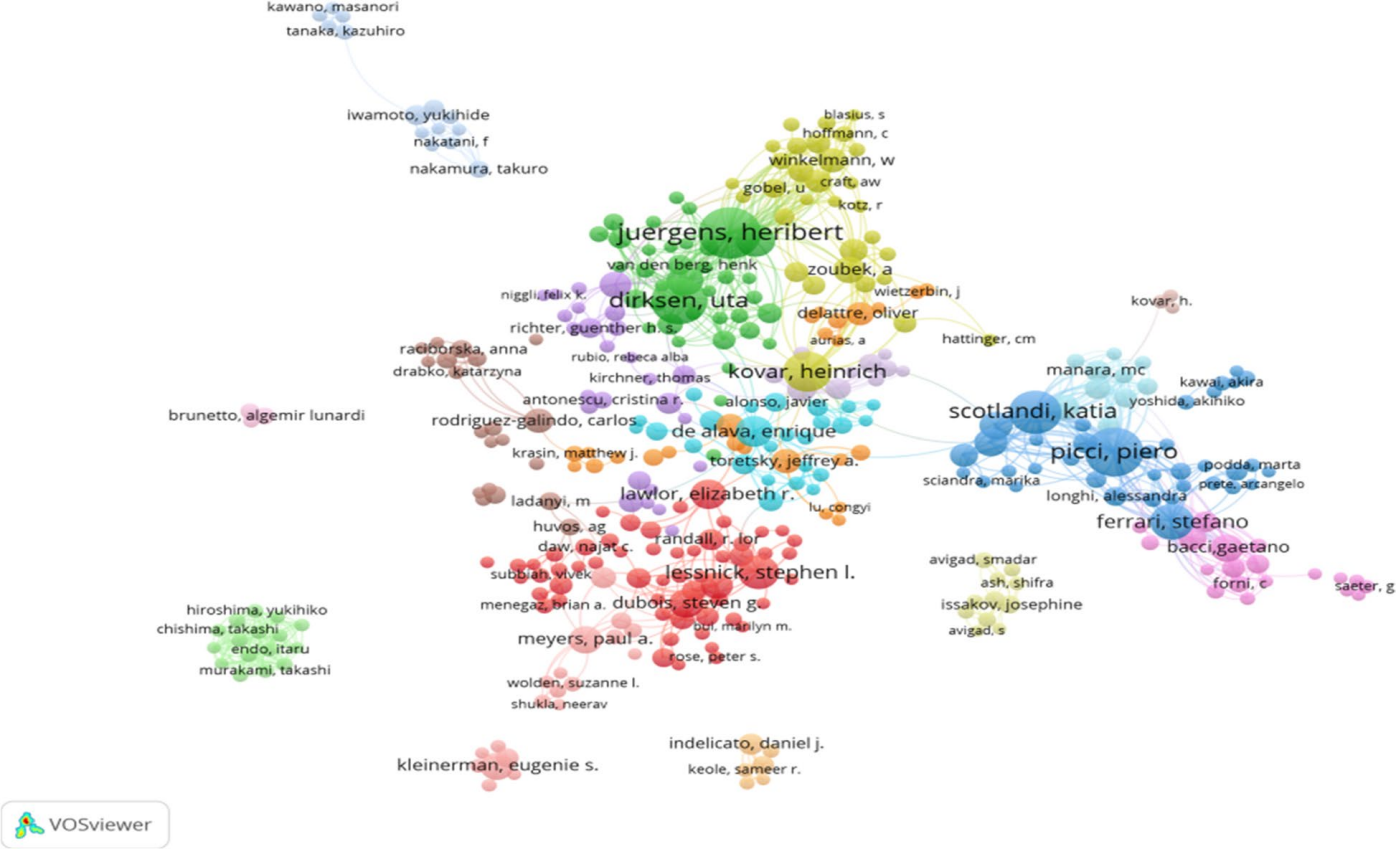
Collaboration of authors on Ewing sarcoma research from 1993 to 2022. Different clusters are represented by different colors

VOSviewer analysis of the authors of published Ewing sarcoma articles revealed that Juergens Heribert published the most articles (84), followed by Picci Piero (83), Dirksen Uta (76), and Scotland Katia (66). Similarly, the number of citations per article is a measure of the quality of the article. Paulussen Michael ranked first with approximately 95.05 citations per article, followed by Juergens Heribert with 55.35 citations per article, and Kovar Heinrich with about 54.68 citations per article (Table 3). The distribution of collaboration revealed the dispersed relationship between these authors, indicating poor collaboration between academics (Fig. 6).

**Table 3:**
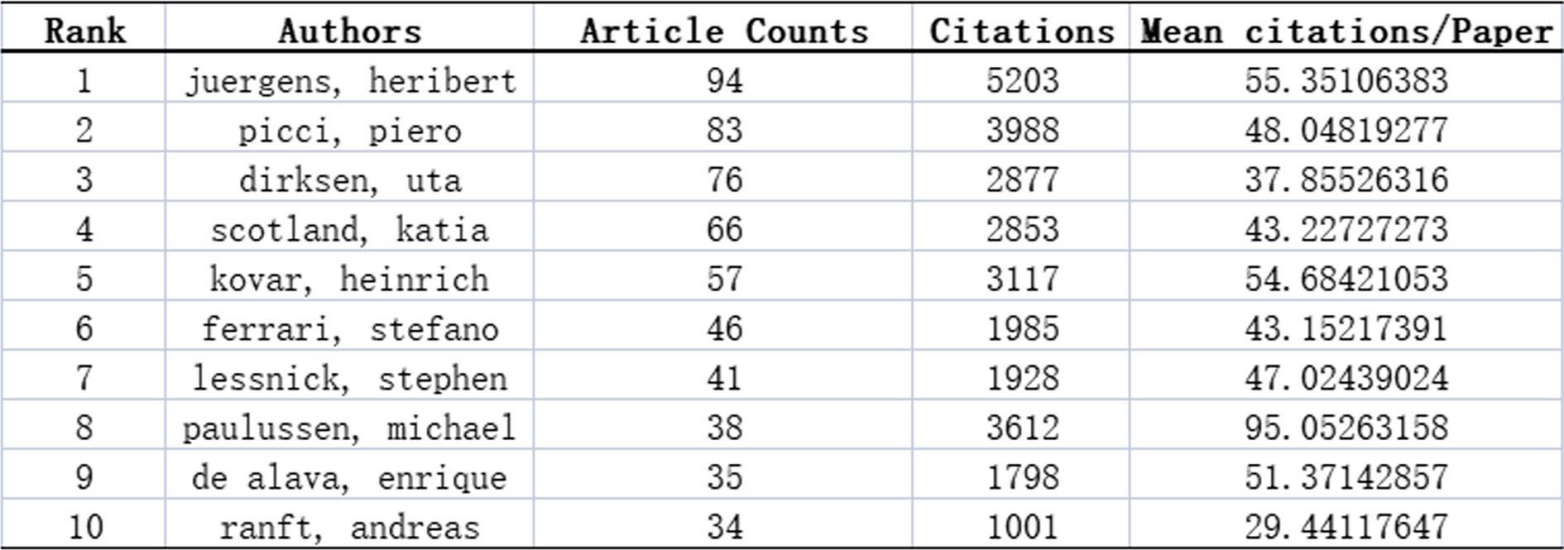
Top 10 authors with the most published studies on Ewing sarcoma from 1993 to 2022

### Analysis of the number of publications

Pediatric Blood & Cancer had the largest number of publications (128), followed by the Journal of Pediatric Hematology Oncology (77) and Cancer research (68). In terms of the average number of citations, the Journal of Clinical Oncology had the largest average number of citations (129.69), followed by Cancer Research (77.97) and Oncogene (59.04). Most of the top 10 journals were in JCR Q1 (Table 4).

**Table 4:**
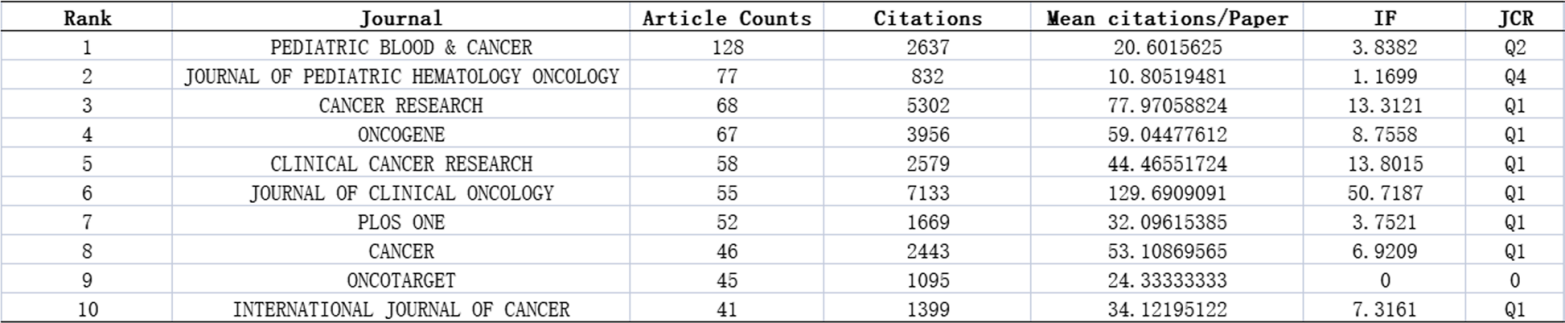
Top 10 publications on Ewing sarcoma from 1993 to 2022

### Funding sponsorship

Ewing sarcoma research has received support from a variety of funding agencies. The top three funding agencies were the USA Department of Health & Human Services, the National Institutes Of Health (NIH) USA, and the National Cancer Institute (NCI) (Table 5).

**Table 5:**
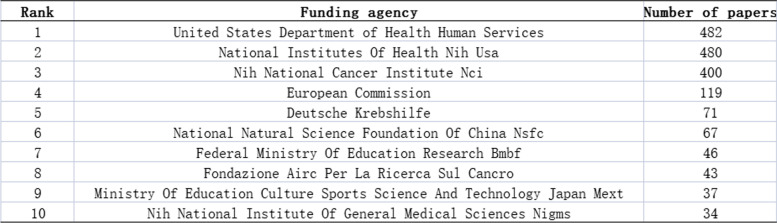
Top 10 funding agencies for Ewing sarcoma research from 1993 to 2022

### Keywords co-occurrence analysis

**Fig. 7:**
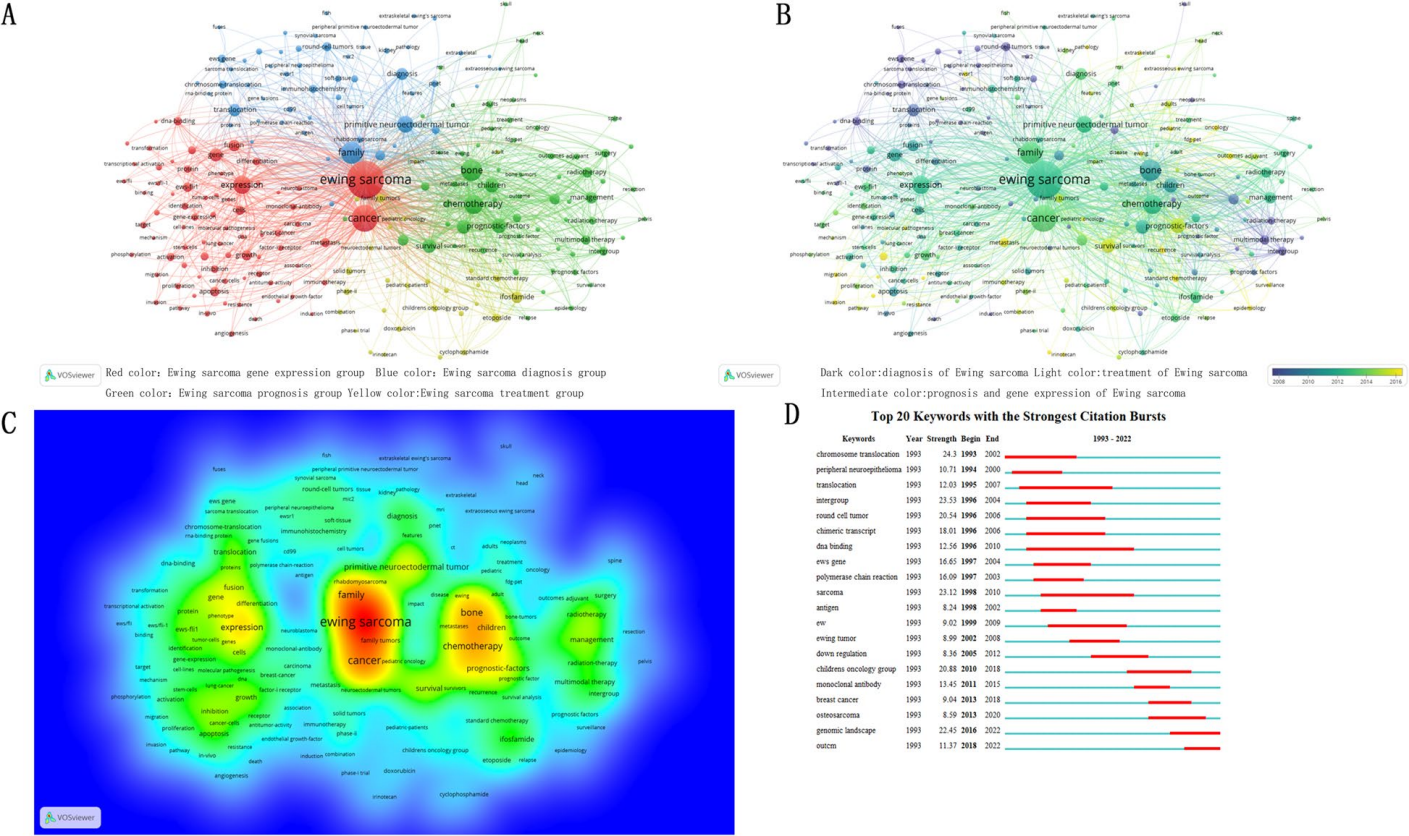
Co-occurrence analysis of global Ewing sarcoma studies from 1993 to 2022 based on the WoSCC database. **A** Mapping of keywords in the research domain. Different clusters are represented by different colors. **B** Mapping time distribution of keywords in the research domain. Different clusters are represented by different colors. **C** Keyword distribution according to the average frequency of occurrence. Red keywords represent the highest frequency. **D** Keywords with the strongest citation in Ewing sarcoma studies

Through VOSviewer, keyword co-occurrence analysis was performed for Ewing sarcoma articles. Keyword co-occurrence analysis refers to the frequency of two keywords appearing in the same article. Similarly, the size of the circles and the thickness of the lines represent the frequency of keyword occurrences and co-occurrences. These keywords were searched in 3016 articles. When the frequency of occurrence was screened to 20, only 226 articles met the conditions. The frequency in the top 10 included “Ewing sarcoma” (*n* = 1789), “tumor” (*n* = 904), "family" (*n* = 602), "bone" (*n* = 580), "chemotherapy" (*n* = 453), "express" (*n* = 425), "primitive neuroectodermal tumor" (*n* = 365), "prognostic factors" (*n* = 286), "children" (*n* = 262), and "survival rate" (*n* = 253) (Fig. 7A). According to the clustering of keyword co-occurrence, the keywords were divided into four groups: Ewing sarcoma gene expression group (red color), Ewing sarcoma diagnosis group (blue color), Ewing sarcoma prognosis group (green color), and Ewing sarcoma treatment group (yellow color) (Fig. 7A). The keywords were measured by the depth of the color of the area. Research work was focused on the diagnosis of Ewing sarcoma before 2010 (dark color), followed by the prognosis and gene expression of Ewing sarcoma from 2010 to 2014 (intermediate color), and the treatment of Ewing sarcoma after 2014 (light color) (Fig. 7B). Meanwhile, the density of the keywords was measured by the color intensity in VOSviewer. The hotspot of this research was often in the red area (Fig. 7C).

### Keywords emergent analysis

CiteSpace was used to analyze the emergence of keywords in Ewing sarcoma. The blue line represented the total timespan, and the red line marked the outbreak period to indicate the start and end of the outbreak. From 1993 to 2010, the outbreak intensity of chromosome translocation was the largest (*n* = 24.3). From 2010 to 2016, the outbreak intensity of the children's oncology group was the largest (*n* = 20.88). After 2016, the outbreak intensity of the genomic landscape was the largest (*n* = 22.45) (Fig. 7D).

### Analysis of the most commonly co-cited articles

The top 10 co-cited articles on Ewing sarcoma are listed in Table 6, with citations ranging from 206 to 485. Delattre O had the highest number of co-citations (*n* = 485), followed by Grier HE and Cotterill SJ with 405 and 400 co-citations, respectively. Most of the articles were published before 2010, and the co-cited articles were all in the Q1 of JCR (Table 6).

**Table 6:**
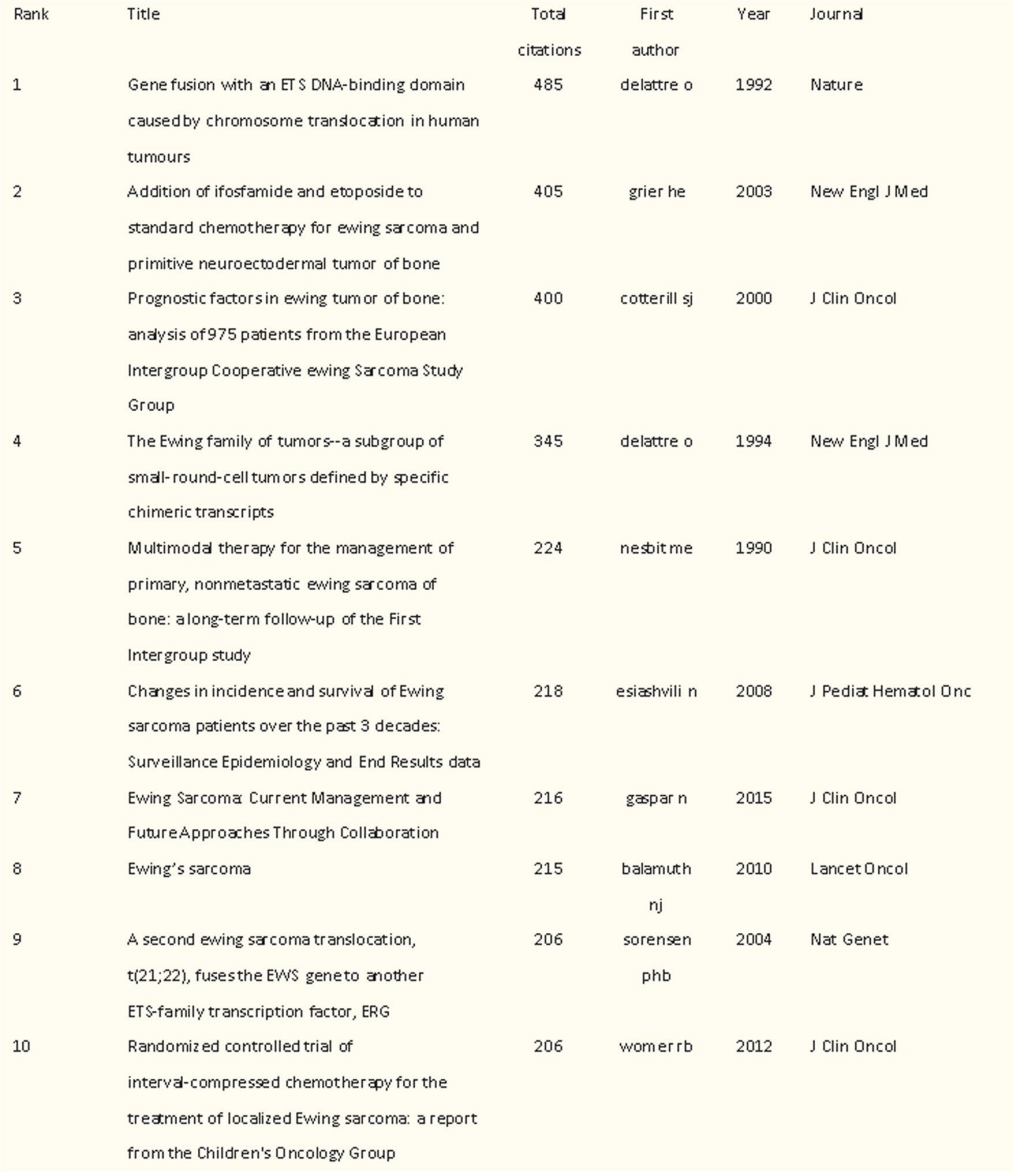
The top 10 co-cited articles on Ewing sarcoma from 1993 to 2022

### Analysis of co-cited reference

**Fig. 8:**
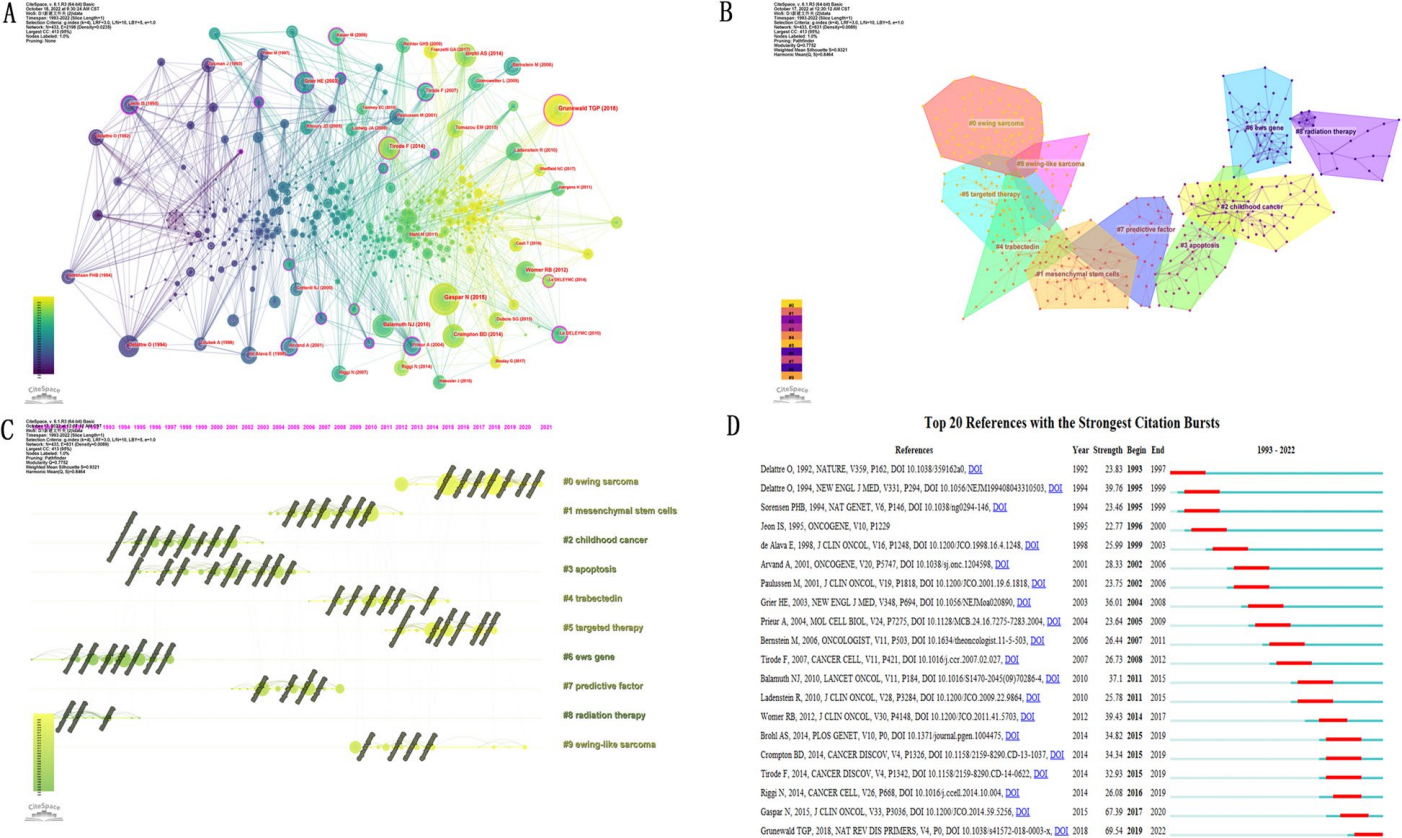
**A** Map of co-cited references in Ewing sarcoma research from 1993 to 2022. The size of each node represents the frequency of an item's occurrence. High centrality nodes with purple rings indicate a turning point or pivot point in the domain. Centrality was used to evaluate the importance of a node in a network, where the centrality corresponded with the number of links passing through the node. **B** Clustering network diagram of co-cited reference in Ewing sarcoma research from 1993 to 2022. Different colors denote different clusters. **C** A timeline view of co-cited clusters of Ewing sarcoma with cluster labels from 1993 to 2022. **D** Top 20 references with the most citation burst in Ewing sarcoma from 1993 to 2022

The analysis of citations is considered an important part of bibliometrics research. Figure 8A illustrates the visual network of co-cited references, consisting of 433 nodes and 2198 links. Each node represents a cited article, and links between nodes indicate the frequency of the same citations. The diameter of a node is proportional to the frequency with which references were cited. The frequency of references to the same article is represented by links between nodes. The node diameter is proportional to the total number of articles co-cited. The developmental stages of a domain can be connected by nodes, where centrality is indicated by a thick purple ring.

The references were divided into 10 groups: Ewing sarcoma, MSC, childhood cancer, apoptosis, trabectedin, targeted therapy, EWS gene, predictive factor, radiation therapy, and Ewing-like sarcoma. After collating the cited literature into a sequence diagram, the treatment of Ewing sarcoma became a hotspot (Fig. 8B and C). Most of the articles were published in JCR Q1 journals, indicating the importance of Ewing sarcoma research (Fig. 8D).

### Distribution of links between journals

**Fig. 9:**
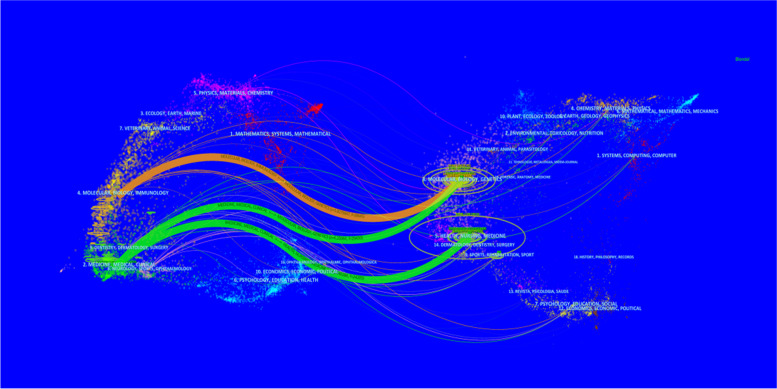
Dual map of journals published on Ewing sarcoma from 1993 to 2022. The cited journal is on the right, the citing journal is on the left, and the straight path represents the citation relationship

The distribution of links between journals was displayed in a dual map overlay of journals, with the citing journals on the left and the cited journals on the right. The described relationships were represented by colored routes connecting them. These labels represented the topics covered by the journals. The primary reference paths were labeled as two green paths and one orange path. The green path illustrated studies published in Molecular/Biology/Genetics and Health/Nursing/Medicine journals that were cited by Medicine/Medical/Clinical journals. The orange route illustrated studies published in Molecular/Biology/Genetics journals that were cited by Molecular/Biology/Immunology journals (Fig. 9).

## Discussion

Ewing sarcoma is the second most common osteosarcoma in children. The most common sites for Ewing sarcoma metastasis are the lungs and bone marrow [1]. The five-year survival rate for patients with localized disease is 60 to 70% as compared with 20 to 45% of patients with metastatic disease [10]. However, the prognosis of Ewing sarcoma has improved over the past decades with the use of multimodal treatment including chemotherapy, surgery, and radiotherapy [11]. However, a large proportion of patients with Ewing sarcoma did not report improved treatment outcomes. Therefore, it is necessary to understand Ewing sarcoma by analyzing past research data. This study reported for the first time the quantitative and qualitative bibliometric analysis of Ewing sarcoma research, covering 3016 research articles from WoSCC. The results revealed that the total number of Ewing sarcoma-related research papers published annually worldwide has gradually increased over the past three decades, reflecting its growing importance in the field of orthopedics.

Of the 3016 articles, the USA contributed 35.9% of the research articles, demonstrating the strong collaboration and highest centrality with other countries. This was followed by Germany, Italy, and China, with lesser cooperation and relatively low centrality. These countries should strengthen international cooperation, especially with leading countries in Ewing sarcoma research. In addition, more than half of the top 10 research institutions are located in the United States, which promotes the progress and development of Ewing sarcoma research. This trend reflects the advancement of Western medical research and the urgency for an effective Ewing sarcoma treatment. NCI, Mem Sloan Kettering Canc Ctr, Inst Curie, and Univ Texas MD Anderson Canc Ctr have the strongest partnerships with other institutions. In terms of fund sponsorship, most of the top 10 fund-sponsoring countries are in Europe and the USA, indicating the high investment in Ewing sarcoma research. China and Japan were also in the top 10 countries sponsored by the fund, indicating the promising research of Ewing sarcoma in Asia.

Juergens Heribert has published the most articles on Ewing sarcoma with 94 articles, followed by Picci Piero (83), Dirksen Uta (76), and Scotland Katia (66). These researchers are mainly situated in Europe and the USA and had worked in orthopedic or oncology departments at their university hospitals. Therefore, strengthening the communication and cooperation among researchers around the world will help the development of Ewing sarcoma research.

The most published journals of Ewing sarcoma are the top journals in the oncology field, including Cancer Research, Clinical Cancer Research, and Journal of Clinical Oncology. The publication of these journal articles on Ewing sarcoma demonstrated that Ewing sarcoma remains a major issue for orthopedic and oncology research. From 1993 to 2002, the top 10 co-cited literature proved that Ewing sarcoma research focused mainly on its treatment.

For keyword co-occurrence, the top 10 keywords were closely related to the age of epidemiology, prognosis, and treatment of Ewing sarcoma. Emergent keywords indicated emerging trends and research frontiers. Through keyword hotspot analysis, the keyword hotspots of Ewing sarcoma focused on chromosome translocation, children's oncology group, and genomic landscape. Delattre O demonstrated that the pathogenesis of Ewing sarcoma is related to the t(11; 22) (q24; q12) chromosomal translocation [12]. It was later reported that Ewing sarcoma has more chromosomal translocations, including EWS/FLI fusion, EWS/ERG fusion, EWS/FEV fusion, EWS/ETV1 fusion, EWS/ETV4 fusion, FUS/ERG fusion, FUS/FEV fusion, and FUS/ETV4 fusion [13]. The children's oncology group is an international research organization dedicated to developing new treatments and cures for cancer in infants, children, adolescents, and young adults [14]. Ewing sarcoma research is important for the research of a new treatment mode to improve the treatment outcome of Ewing sarcoma. Studies have reported that the most common mutations in Ewing sarcoma are the loss of function of STAG2, TP53, and CDKN2A genes [15]. Moreover, some studies have also indicated that PIK3R1, NOTCH1, and CREBBP are common in recurrent Ewing sarcoma [16]. The functional changes of TP53, PMS2, and RET genes are also important factors in inducing Ewing sarcoma [17].

The timeline of the cluster map of co-cited literature revealed that the treatment of Ewing sarcoma recently became a research hotspot. There are many treatment methods for Ewing sarcoma, including radiotherapy, surgery, chemotherapy, and targeted drug therapy. Ewing sarcoma is sensitive to radiation therapy, and preoperative neoadjuvant radiotherapy can shrink the tumor and reduce the risk of surgery [18, 19]. For metastasized tumors, radiotherapy can be used for treatment [20]. Adjuvant chemotherapy with surgery or radiotherapy can significantly improve the survival rate of patients with Ewing sarcoma [21]. Drugs, including ecteinascidin, cabozantinib, and regorafenib, have reported good efficacy in the treatment of Ewing sarcoma [22–24]. Likewise, the advancement of radiotherapy and targeted therapy has significantly improved the survival rate of pediatric and adolescent cancer patients.

## Limitations

Some limitations of this study should be addressed. WoSCC database is considered to be the most important data source for bibliometric analysis, so the only database used in this study. Hence, some studies might have been missed. In addition, only articles and reviews, as well as English-language publications, were used in the analysis, which might have led to some bias. Furthermore, the author's affiliation could not be fully established. Some authors might have duplicated names, or the same author could be from a different institution. That said, this study established research focus and emerging trends in Ewing sarcoma.

## Data Availability

All data generated or analyzed during this study are included in this published article.
